# From polyclonal to monoclonal: de novo sequencing of goat antibodies for a standardized ApoA-I immunoturbidimetric assay

**DOI:** 10.1186/s13036-026-00697-y

**Published:** 2026-05-12

**Authors:** Lulai Xu, Qitao Song, Ming Zhu, Wei Zhao, Qiang Li, Sai Li, Bohong Li, Linzhu Li, Bin Ma, Zhengzhong Song, Xin Yang, Yun Xie, Weijing Yi, Jing Wang

**Affiliations:** 1 Animal Disease Prevention and Green Development Key Laboratory of Sichuan Province, College of Life Science, Sichuan University, Chengdu, 610064 China; 2 Department of Laboratory Medicine, Northwest Women’s and Children’s Hospital, Xi’an, Shaanxi 710000 China; 3 Zybio Inc., Chongqing Municipality, 400039 China; 4 Chongqing Essence Biological Engineering Co., Ltd., Chongqing, 400082 China; 5 KuaiXu (ShangHai) Biotechnologies Co., Ltd., Shanghai, 201315 China; 6 Lianyungang 149 Hospital, 127 haitang North Road, Lianyun District, Lianyungang City, Jiangsu province 222000 China

**Keywords:** Apolipoprotein A-I, De novo sequencing, Monoclonal antibody, Immunoturbidimetric assay, Standardization

## Abstract

Antibodies serve as essential recognition elements in in vitro diagnostics (IVD), yet reliance on polyclonal antibodies (pAbs) introduces significant challenges related to batch-to-batch consistency. To address this, we employed a *de novo* sequencing platform, which identified 15 candidate monoclonal antibody (mAb) sequences. From these, four distinct mAb mixtures were formulated and evaluated. Using an immunoturbidimetric assay to quantify apolipoprotein A-I (ApoA-I) in 105 human serum samples, the performance of these mixtures was compared to that of a conventional pAb reagent. The four-mAb mixture demonstrated excellent correlation with the pAb assay (Passing-Bablok: y = 1.0536x-0.0748 g/L; r^2^ = 0.994). This optimized cocktail successfully replaced the pAb reagent, matching or exceeding its performance in key analytical parameters including linearity, sensitivity, and precision. Our work thus establishes a practical pathway for converting heterogeneous pAbs into defined, recombinant antibody reagents suitable for standardized IVD applications.

**Supplementary Information:**

The online version contains supplementary material available at 10.1186/s13036-026-00697-y.

## Introduction

Antibodies are indispensable recognition elements in vitro diagnostics (IVD). However, the industry’s dependence on polyclonal antibodies (pAbs) poses a significant challenge to achieving robust assay standardization. Although pAbs offer broad epitope coverage, their composition is inherently heterogeneous. This variability stems from differences in immunogens, animal hosts, and immunization protocols, leading to substantial batch-to-batch inconsistencies [1, 2]. This batch-to-batch inconsistency directly compromises the long-term reliability and regulatory compliance of diagnostic tests, creating an urgent need for defined, reproducible alternatives.

To combine broad epitope recognition with batch-to-batch consistency, monoclonal antibody (mAb) cocktails present a promising alternative to polyclonal reagents [3]. Their development, however, is constrained by a fundamental shortcoming of standard discovery platforms such as phage display [4] or single B-cell cloning [5]. These methods are designed to prioritize the acquisition of antibody gene sequences. Consequently, the direct selection of clones based on superior binding function—particularly those within a polyclonal response that exhibit the highest affinity and specificity—becomes secondary. The functional validation of selected sequences remains a separate, subsequent step, which can allow the most effective antibodies to be overlooked during initial screening [6].

To avoid the limitations of conventional monoclonal antibody discovery platforms, we have refined the *de novo* sequencing workflow by incorporating the function-based screening step. Specifically, we perform direct sequencing of antigen-antibody immunoprecipitation complexes, enabling the *de novo* elucidation of corresponding amino acid sequences from mass spectrometry data. This strategy has been validated in studies using human serum, successfully identifying antibodies with neutralizing activity [7, 8]. The present work aims to adapt this methodology for industrial-grade polyclonal antibody production, with the goal of transforming them from biological preparations subject to significant batch-to-batch variation into stable, well-defined, and renewable recombinant reagents.

To test this hypothesis, we focused on Apolipoprotein A1 (ApoA-I), a well-established biomarker for cardiovascular risk assessment. As the principal component of high-density lipoprotein (HDL), ApoA-I is central to reverse cholesterol transport, and its accurate quantification is essential for clinical diagnostics [9, 10]. Commonly used immunoturbidimetric and ELISA methods for ApoA-I [11], however, are susceptible to the limitations of pAb reagents, underscoring the need for improvement.

Here, we report a comprehensive workflow to re-engineer a goat-derived anti-ApoA-I pAb into a defined recombinant mAb cocktail. Our strategy centers on a critical functional step: immunoprecipitation of the target antigen to isolate the most relevant antibody subset. By subjecting this functional fraction to *de novo* sequencing, we identified the key sequences responsible for antigen recognition. We then recombinantly produced these antibodies, formulated an optimal cocktail, and rigorously evaluated its performance in a clinical immunoturbidimetric assay. The resulting four-mAb cocktail not only matched but exceeded the performance of the original pAb, demonstrating exceptional correlation with clinical samples and superior batch-to-batch consistency. This study establishes a generalizable strategy for overcoming the standardization bottleneck in the IVD industry.

## Materials and methods

### Ethical statement and animal immunization

All animal immunization and serum collection procedures were conducted under a service agreement by Shandong Aileke Biotechnology Co., Ltd., and were approved by the company’s Animal Care and Use Committee (Approval No. 2024-12-16-93).All procedures complied with the institution’s Animal Welfare Guidelines. Antiserum was collected when the antibody titer reached or exceeded 1:32.

### Functional enrichment of antigen-specific polyclonal antibodies

Anti-ApoA-I antibodies were isolated from goat antiserum by immunoprecipitation with ApoA-I antigen. Immune complexes were precipitated with 4% PEG 6000, centrifuged at 10,000 × g for 30 min at 4 °C, and resuspended in PBS (pH 7.4) for proteomic analysis.

### RNA sequencing and antibody gene repertoire analysis

Total RNA was extracted from peripheral blood mononuclear cells (PBMCs) using a FastPure RNA Kit (Vazyme Biotech Co., Ltd, Nanjing, China, Cat# RC112). First-strand cDNA was synthesized with the HiScript-TS 5´/3´ RACE Kit(Vazyme Biotech Co., Ltd, Nanjing, China, Cat# RA101), followed by amplification of antibody variable regions via 5´ RACE PCR (68 °C annealing). The PCR products were sequenced on a NovaSeq 6000 platform, and the raw reads were assembled into variable region sequences using the IMGT germline database.

### De novo antibody sequencing and heavy-light chain pairing

#### Sample preparation for mass spectrometry

Antigen-enriched pAbs were digested using complementary approaches to maximize sequence coverage. For in-solution digestion, reduced and alkylated pAbs were subjected to multi-enzyme digestion (Trypsin, LysC, AspN, Chymotrypsin, Pepsin). In parallel, pAbs were separated under native and non-reducing conditions by SDS-PAGE [12]. Protein bands were excised, subjected to in-gel reduction, alkylation, and tryptic digestion.

#### LC-MS/MS analysis

Digested peptides were analyzed using an Orbitrap Eclipse Tribrid mass spectrometer (Thermo Fisher Scientific). Chromatographic separation was performed on a 15-cm EVOSep column (#EV1106). MS data were acquired in a data-dependent mode, surveying m/z 400–2000 at a resolution of 60,000. MS/MS fragmentation was performed using HCD at a fixed 30% normalized collision energy and a resolution of 7,500. Dynamic exclusion was set to 15 s. For each MS run, approximately 1 µg of digested peptides was injected. Mobile phase A consisted of water with 0.1% formic acid, and mobile phase B was acetonitrile with 0.1% formic acid. A predefined 88-minute LC gradient in the EVOSep was used.

A quality control (QC) sample (a pool of all digests) was analyzed at the start and after every 10 experimental runs to monitor instrumental performance. Only high-confidence peptide-spectrum matches (PSMs) with a Novor confidence score ≥ 75 and a false discovery rate (FDR) < 1% were retained for subsequent analysis.

#### Database construction and de novo sequence assembly

A custom protein sequence database was constructed by translating merged NGS reads (R1 and R2) with reference to the IMGT germline database, followed by deduplication.

De novo sequencing was performed using Novor software as previously described ^[6^. Peptide sequences with a confidence score ≥ 75 were assembled into contigs via a graph-based algorithm that maximizes “mass block” overlap. Contigs were aligned to germline sequences, and those fully covering a complementarity-determining region (CDR) plus flanking residues were retained. These high-quality contigs were searched against the MS/MS data using Novor.Cloud, grouped by CDR region, and prioritized based on length, PSM coverage, and peptide overlap. A final shortlist of CDR sequences was curated through manual inspection to correct subtle sequencing errors and resolve ambiguities using long peptides spanning multiple CDRs. The selected CDR1, CDR2, and CDR3 sequences were then assembled into full-length variable (V) regions.

#### Heavy-light chain pairing and validation

Heavy-light chain pairing was determined by integrating spectral similarity and co-elution data from non-reducing and native gel separations. Novor.Cloud was used to identify PSMs for each chain, and MaxQuant (v2.1.3) was used to extract peptide peak areas across fractions. A pairwise similarity score between heavy and light chains was computed based on their peptide co-elution profiles.

The top 85 heavy-light chain pairs, ranked by their spectral similarity scores, were selected for recombinant expression. Antigen-binding specificity of these pairs was confirmed via ELISA using ApoA-I-coated plates, thereby validating the bioinformatic predictions.

### Recombinant antibody and antigen expression

The genes encoding the engineered antibodies (based on a goat Fc backbone) and the C-terminal 6×His-tagged human ApoA-I antigen were cloned into the pcDNA3.1 vector, and all constructs were verified by DNA sequencing. Proteins were transiently expressed in CHO cells cultured in serum-free medium for six days [13]. The antibodies were purified by Protein G affinity chromatography, and the ApoA-I antigen was purified using a Ni Sepharose Excel column (Cytiva). Finally, the antibodies were dialyzed into 20 mM PBS (pH 7.4), and the antigen was dialyzed into 20 mM Tris, 150 mM NaCl (pH 8.0).

### Immunoturbidimetric assay development and evaluation

#### Reagents and instruments

The immunoturbidimetric assay [14] for ApoA-I was developed using monospecific goat anti-human ApoA-I polyclonal antiserum (Zybio Inc., China) as the starting material for recombinant mAb generation. The optimized four-mAb cocktail was formulated for assay use. The assay buffer was phosphate-buffered saline (PBS; 137 mM NaCl, 9.8 mM Na₂HPO₄, 2.0 mM NaH₂PO₄, pH 7.4) containing 48 g/L polyethylene glycol 8000 and 0.1% (v/v) Tween 20. The antiserum and mAb cocktail were diluted offline in a Tris buffer (200 mM Tris-HCl, 498 mM NaCl, 7.7 mM NaN₃, pH 7.5). Sample diluent was PBS. All chemicals were reagent grade, and all buffers were filtered through a 0.22-µm membrane before use.

Assay performance was evaluated on a Beckman Coulter AU680 chemistry analyzer and a Zybio EXC2000 chemistry analyzer.

#### Assay validation procedures

##### Calibration and quality control

The assay was calibrated using purified recombinant human ApoA-I antigen. Five-point calibration curves were established for both the mAb cocktail (0, 0.75, 1.12, 1.56, 2.05 g/L) and the original pAb reagent (0, 0.67, 1.10, 1.66, 2.10 g/L). Two levels of commercial quality control materials, QC1 and QC2 provided with the ApoA-I assay kit from Zybio Inc., Chongqing, China, were analyzed in duplicate per run, with assay validity requiring all controls to fall within the manufacturer’s specified ranges (± 2 SD).

##### Linearity

The analytical measurement range was verified using two human serum samples: a high-concentration patient sample (ApoA-I level approximately 2.8 g/L) and a low-concentration patient sample (ApoA-I level approximately 0.5 g/L). These two samples served as the undiluted endpoints. Intermediate concentration points were prepared by mixing the high- and low-concentration sera in defined ratios (e.g., 1/8 high + 7/8 low, 2/8 high + 6/8 low, etc.) to generate a dilution series. The observed concentrations were plotted against the expected values and analyzed by linear regression. The assay was considered linear within the range if the coefficient of determination (R²) was ≥ 0.990.

##### Precision assessment

Precision was evaluated through two complementary experiments. First, for comparison between the polyclonal antibody (pAb) and mixed monoclonal antibody (mAb) cocktail reagents, three independent batches of each reagent were prepared. Low-, medium-, and high-concentration control samples were measured in 10 replicates per batch. Intra-batch precision was calculated as the coefficient of variation (CV) from the 10 replicates within a single run, and inter-batch precision was calculated across the three batches. Second, to verify agreement between the mAb cocktail and the commercial Roche reference assay, medium-concentration controls were used, with 10 replicates within one run.

##### Stability testing

Short-term stability of the mAb cocktail and the pAb reagent was evaluated under storage at room temperature (approximately 25 °C). Four quality control samples (QC1, QC2, L value, and M value) were measured on days 1, 5, 6, 9, 12, 16, 20, and 28. The percent deviation from the initial (day 0) measurement was calculated for each time point.

##### Interference testing

The potential interference of triglycerides (5–20 g/L), hemoglobin (2.5–10 g/L), and vitamin C (0.25–1 g/L) was evaluated by spiking these substances into patient samples at five physiologically relevant concentrations. Interference was deemed clinically insignificant if the measured bias was less than ± 10% compared to the unspiked baseline.

##### Method comparison

The clinical performance of the novel mAb-based assay was compared against a commercially available reference method, the Roche Apolipoprotein A1 assay kit (Roche Diagnostics, Germany), using 50 residual clinical serum samples. The agreement between methods was assessed by Passing-Bablok regression [15] and Bland-Altman analysis [16].

##### Ethical statement for human samples

The study involved 250 de-identified residual serum samples obtained from the Second Affiliated Hospital of Chongqing Medical University. The protocol was approved by the Ethics Committee of the Second Affiliated Hospital of Chongqing Medical University (Approval No. 2025 − 268). Informed consent was waived by the committee because the samples were residual specimens from routine laboratory testing and were used anonymously. All procedures complied with the Declaration of Helsinki.

### Statistical analysis

The sample size for the method comparison was calculated a priori using PASS 2021 software (NCSS, LLC). To robustly demonstrate a high correlation (R² ≥ 0.98) between our novel mAb-based assay and the reference method, against a null hypothesis of R² = 0.95 with 90% power and an alpha of 0.05, a minimum of 98 samples was required. We prospectively enrolled 105 clinical samples to account for potential exclusions and to ensure precise estimates in the subsequent Bland-Altman analysis.

All statistical analyses were performed using GraphPad Prism (Version 10.5.0; GraphPad Software, San Diego, CA, USA). The distribution of all quantitative data was assessed for normality using the Shapiro-Wilk test.

Method Comparison: The agreement between our ApoA-I assay and the two comparator methods was evaluated using Passing-Bablok regression and Bland-Altman difference plots. Bland-Altman analysis was used to quantify the mean bias and 95% limits of agreement.

Outlier Handling: Potential outliers were identified using the Grubbs’ test (alpha = 0.05) but were retained in the primary analysis to reflect the assay’s performance in a realistic clinical setting.

## Results

### Integrated platform for antibody discovery from polyclonal repertoire

**Fig. 1:**
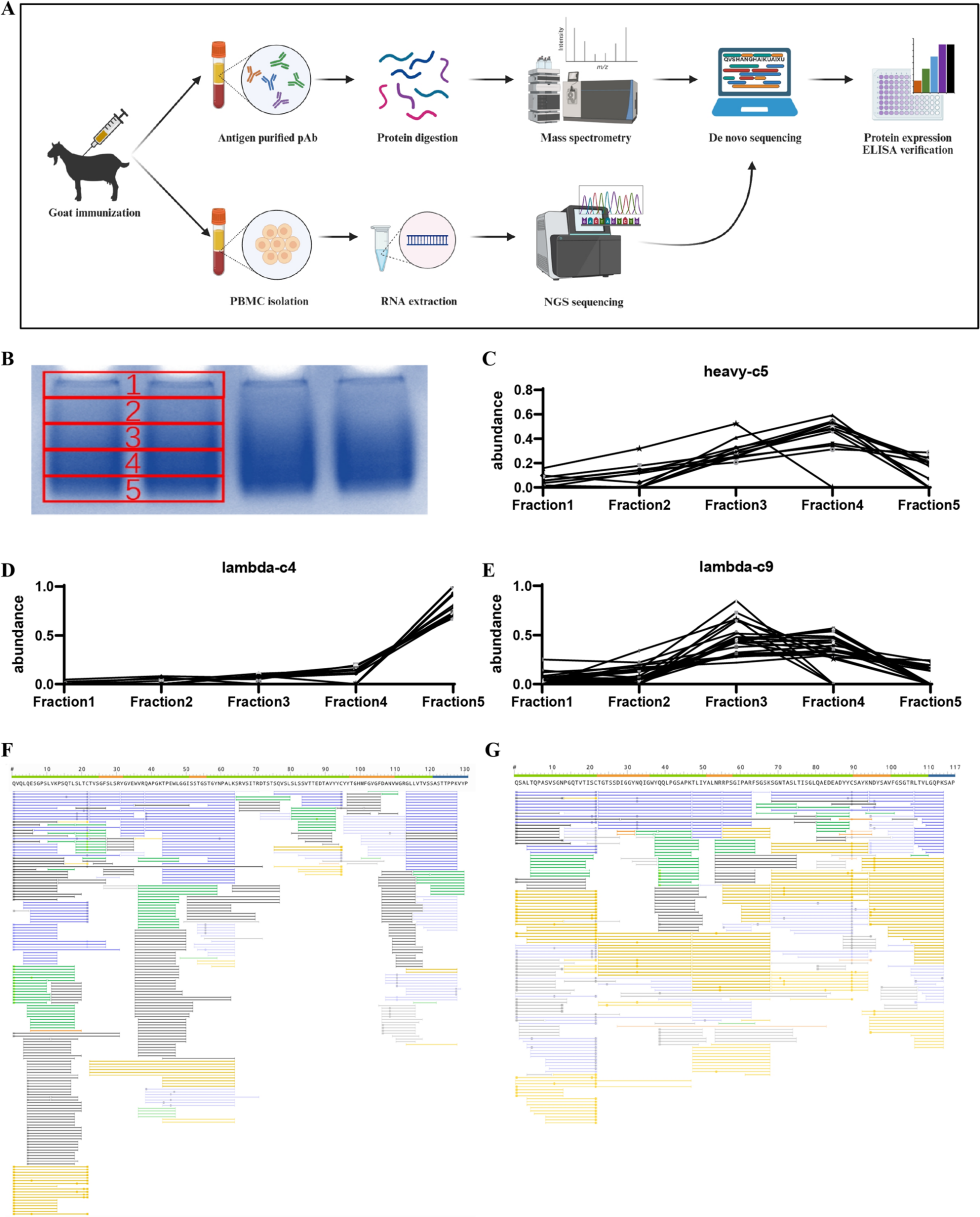
An integrated pipeline for de novo antibody discovery and validation from immunized goats. (**A**) Schematic overview of the discovery workflow. Plasma and PBMCs from immunized goats were processed in parallel. Antigen-specific pAbs from plasma were subjected to multi-protease digestion and high-resolution mass spectrometry for *de novo* protein sequencing, while PBMC-derived RNA was used for next-generation sequencing (NGS) of the antibody repertoire. (**B**-**E**) Gel-based heavy-light chain pairing and functional validation. (**B**) Native gel separation of pAbs, with the lane excised into five consecutive segments for analysis. (**C**-**E**) Co-elution profiling confirmed the native pairing between heavy chain H5 and light chain L9 (**C**, **E**), while disproving the pairing with L4 (D). (**F**-**G**) Validation of sequenced antibodies. Representative MS/MS sequence coverage of two identified antibodies: the heavy chain H5 (**F**) and its paired light chain L9 (**G**). Each colored line represents a confidently identified peptide. The CDR1, CDR2, and CDR3 regions are highlighted in orange

We established a comprehensive platform to convert a functional polyclonal antibody (pAb) into a defined mAb cocktail, combining next-generation sequencing (NGS) with de novo protein sequencing. As outlined in Fig. 1A, the process began with the isolation of plasma and PBMCs from immunized goats. Antigen-specific pAbs were affinity-purified from plasma and subjected to multi-protease digestion (trypsin, LysC, AspN, chymotrypsin, pepsin) for mass spectrometry (MS) analysis. Concurrently, RNA extracted from PBMCs was used to construct an NGS library of the antibody repertoire, yielding 882,176 antibody sequences from 1,373,350 cDNA reads.

### De novo sequencing and heavy-light chain pairing

High-resolution MS analysis of the digested pAbs enabled de novo sequence determination. Cross-referencing MS data with the NGS database identified high-confidence sequences, which were clustered based on CDR3 homology. This integrated approach yielded 25 heavy-chain, 1 kappa-chain, and 33 lambda-chain clusters. To accurately pair heavy and light chains, we performed native and non-reducing gel electrophoresis followed by in-gel digestion and MS analysis of consecutive gel segments (Fig. 1B). The digested peptides were analyzed by MS, and the MS data were searched against the list of potential antibodies. The high similarity between the profiles of Heavy-c5(Fig. 1C) and Lambda-c9(Fig. 1E) suggested they form a native pair, unlike Heavy-c5 and Lambda-c4(Fig. 1D).

### Expression and functional screening of recombinant antibodies

A total of 85 candidate antibodies were recombinantly expressed and screened for antigen binding by ELISA. Among these, 19 exhibited high affinity for ApoA-I, with 7 showing affinity comparable to or greater than the original pAb (Fig. S1). Based on the diversity of heavy chains, 15 antibodies with high affinity were selected for large-scale recombinant expression. To identify antibodies with complementary epitope recognition, we performed pairwise synergy screening. The 15 purified mAbs were systematically combined in pairs, and their binding signals for high-, medium-, and low-concentration ApoA-I samples were evaluated. Six mAbs (designated 1# to 6#) that, in various combinations, produced robust and dose-dependent signals were selected. These six mAbs were then formulated into a base cocktail (referred to as Group A in subsequent optimization) for systematic evaluation.

### Optimization of the mAb cocktail via systematic combination screening

**Fig. 2:**
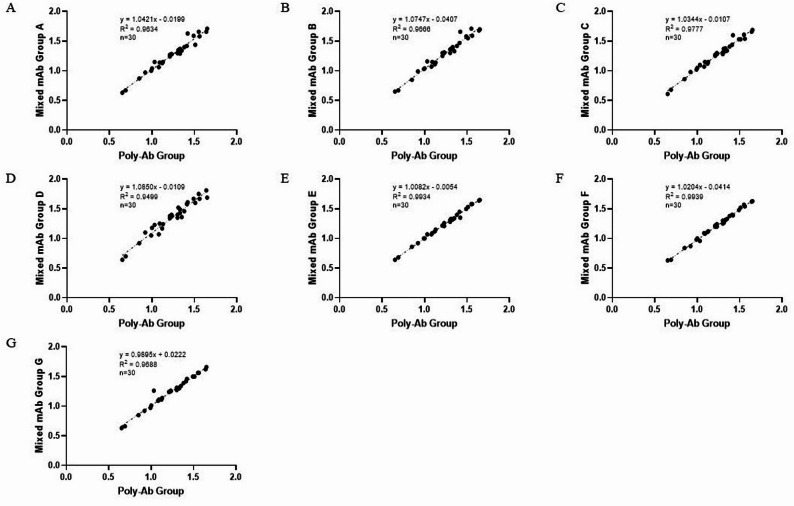
Functional deconvolution of the antibody cocktail identifies critical contributors through “leave-one-out” screening. The full six-antibody cocktail (Group **A**) and six derivative mixtures, each lacking a single antibody (Groups **B**–**G**), were tested for clinical correlation (R²) against a polyclonal reference standard

From the 85 predicted heavy-light chain pairs, ELISA screening identified 19 with high binding affinity to ApoA-I, of which 7 exhibited affinity comparable to or better than the original polyclonal antibody (Fig. S1). Based on heavy-chain sequence diversity, 15 high-affinity antibodies were selected. Pairwise synergy screening using high-, medium-, and low-concentration ApoA-I samples then identified six antibodies (designated 1#–6#) that consistently produced dose-dependent signals. To identify the optimal antibody combination among these six, we systematically evaluated mixtures with sequential antibody removal (Table S1). The full six-antibody cocktail (Group A) showed a baseline correlation of R² = 0.9634 against the pAb-based reference. Testing against 30 clinical samples revealed that removing mAb 4# or 5# significantly improved correlation (R² = 0.9934–0.9939), whereas removing mAb 3# substantially reduced performance (R² = 0.9499). Removal of antibody #6 yielded an intermediate R² (0.9688) that was not clearly antagonistic or essential (Fig. 2). These results indicated antagonistic effects from mAbs 4# and 5#, and a critical contribution from mAb 3#. This data-driven optimization underscores that an effective mAb cocktail requires not only high-affinity binders but also synergistic epitope coverage. Accordingly, a refined panel of four combinations—Group 1 (1#+3#), Group 2 (1#+2#+3#), Group 3 (1#+3#+6#), and Group 4 (1#+2#+3#+6#)—was advanced to clinical validation.

### Clinical evaluation of the optimized antibody cocktail

**Fig. 3:**
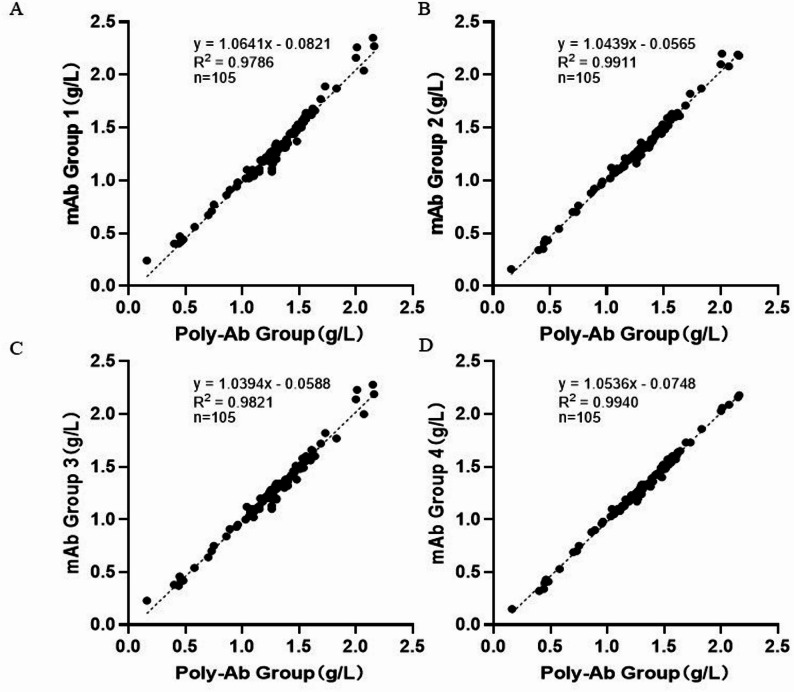
Clinical correlation of optimized monoclonal antibody cocktails against a polyclonal reference. Linear regression analysis of ApoA-I measurements from 105 clinical serum samples, comparing four candidate monoclonal antibody cocktails with the reference polyclonal antibody reagent. The dashed lines represent the linear regression curves for each group: Group 1 (mAb 1#+3#), Group 2 (mAb 1#+2#+3#), Group 3 (mAb 1#+3#+6#), and Group 4 (mAb 1#+2#+3#+6#). The corresponding regression equations and coefficients of determination (R²) are displayed for each group

Based on the synergy screening results that identified mAbs 4# and 5# as antagonistic, we excluded these clones. The four most promising candidate cocktails—designated Group 1 (1#+3#), Group 2 (1#+2#+3#), Group 3 (1#+3#+6#), and Group 4 (1#+2#+3#+6#)—were subsequently validated using 105 fresh clinical samples (Fig. 3). For the linear regression analysis, the R² values for the four groups were 0.9786, 0.9911, 0.9821, and 0.9940, respectively. The slopes and intercepts of the linear regression analysis for the four groups were as follows: 1.0641, -0.0821; 1.0439, -0.0565; 1.0394, -0.0588; and 1.0536, -0.0748.

**Fig. 4:**
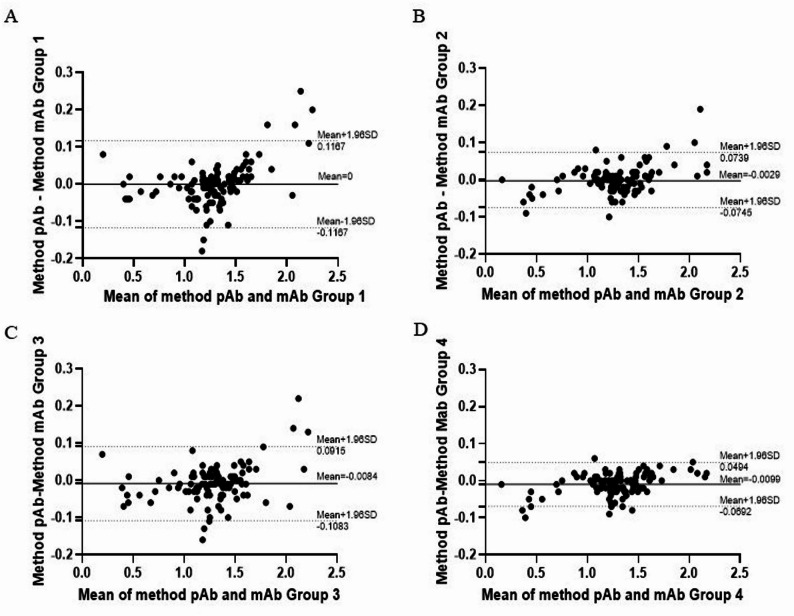
Analytical agreement of monoclonal antibody cocktails assessed by Bland-Altman analysis. The difference between the four monoclonal antibody cocktails and the polyclonal reference method is plotted against their average measured ApoA-I concentration for 105 samples. The solid central line represents the mean difference (bias), and the dashed lines indicate the 95% limits of agreement

The Bland-Altman analysis (Fig. 4) demonstrated excellent agreement between the four-novel monoclonal ApoA-I reagent kits (Groups 1–4) and conventional polyclonal kits. The mean differences were remarkably small: 0, 0.0029, 0.0084, and 0.0099 g/L for Groups 1–4 respectively, with all 95% confidence intervals falling well within our pre-defined clinical equivalence margin of ± 0.15 g/L. The 95% limits of agreement were similarly tight, ranging from − 0.1167 to 0.1167 for Group 1 to -0.0692 to 0.0494 for Group 4. Notably, 98.1% of all measurements across monoclonal kits remained within clinical acceptability limits. Among the four cocktails, Group 4 (the four-mAb mixture) demonstrated the best overall agreement, although some variability was observed at extreme concentration ranges for several groups (Fig. 4), particularly at high antigen concentrations where simpler cocktails showed increased variability.

### Analytical performance of the four-mAb cocktail reagent

**Fig. 5:**
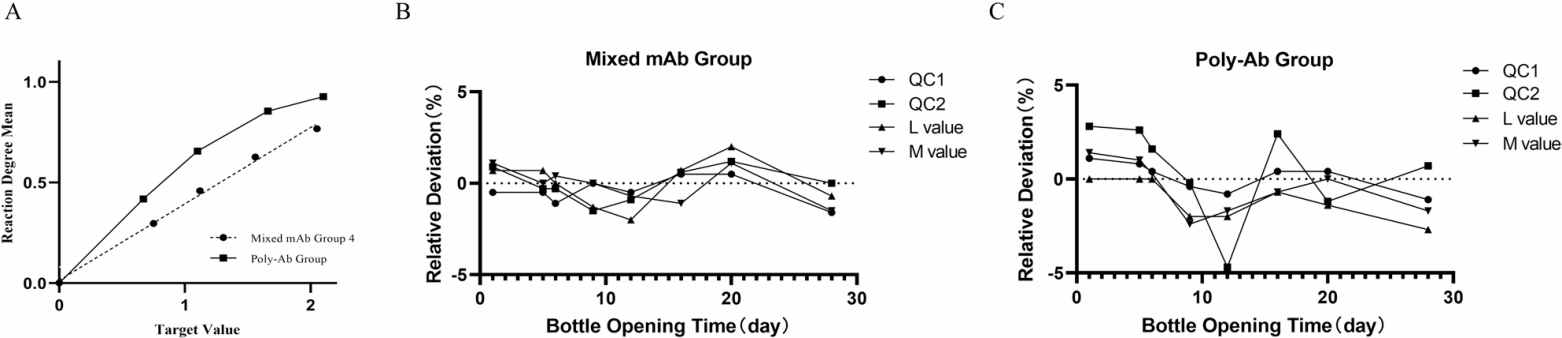
Analytical performance and stability of the optimized monoclonal antibody (mAb) cocktail reagent. (**A**) Standard curves of the monoclonal (mAb Group 4) and polyclonal (pAb) reagents. (**B**, **C**) Short-term stability evaluation of the (**B**) mAb and (**C**) pAb reagents over 30 days, showing the percent deviation from the initial measurement. QC1 and QC2 denote quality control samples at low and medium concentrations, respectively; L value and M value refer to the low-concentration and medium-concentration controls used for precision assessment

The optimized four-mAb cocktail (Group 4: mAbs 1#+2#+3#+6#) was used for all analytical performance evaluations.As shown in Fig. 5A, the standard curves of the pAb and mAb groups were constructed using reference standards. The standard curve of the mAb group was linear, while that of the pAb group was nonlinear.

The linear range of the mAb group was 0.4–2.5 g/L, which was broader than that of the pAb group (0.4–2.2 g/L) (Table S4 and Fig. S2). Furthermore, the mixed mAb reagent met the anti-HOOK performance criterion at a concentration of 3.3 g/L (data not shown).

In terms of precision, the mixed mAb reagent demonstrated excellent precision. The intra-batch variability of the mixed mAb reagent was slightly better than that of the pAb group (mAb high, medium, and low values were 0.94%, 0.84%, and 0.76%, respectively, while pAb high, medium, and low values were 1.18%, 0.96%, and 0.93%, respectively). Furthermore, the inter-batch variability of the mixed mAb reagent was significantly better than that of the pAb group (mAb high, medium, and low values were 1.0%, 1.61%, and 1.92%, respectively, while pAb high, medium, and low values were 1.87%, 2.83%, and 5.85%, respectively) Detailed results are presented in Table S3.

Stability testing revealed that the mixed mAb reagent exhibited excellent short-term stability, with deviations less than 2% over 30 days. In comparison, the pAb reagent showed deviations less than 5% over the same period (Fig. 5B and C).

The interference resistance of both reagents was evaluated and summarized in Table S2. Both the mixed mAb reagent and the pAb reagent demonstrated robust resistance to interference from 5 g/L triglyceride, 1 g/L ascorbic acid, and 10 g/L hemoglobin.

**Fig. 6:**
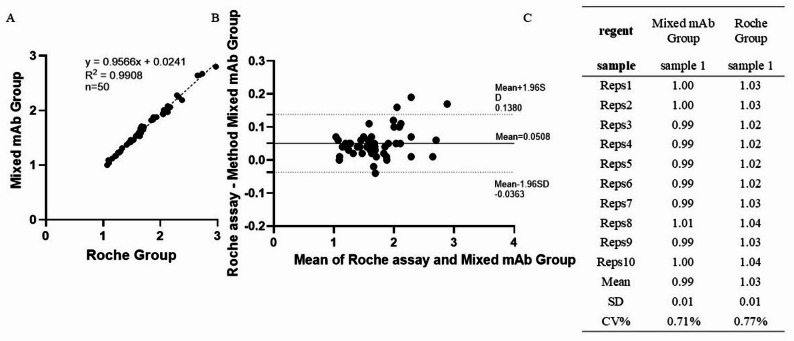
Method comparison and precision of the monoclonal antibody (Mixed mAb) reagent against the Roche clinical assay. (**A**) Correlation analysis of ApoA-I measurements from 50 clinical samples. The solid line represents the linear regression fit (R² = 0.9908). (**B**) Bland-Altman difference plot. The solid line indicates the mean difference (bias), and the dashed lines represent the 95% limits of agreement (mean difference ± 1.96 SD). (**C**) Precision testing results

To evaluate the accuracy of the mixed mAb reagent, it was compared with Roche Apolipoprotein A1 assay kit. Fifty samples were tested simultaneously, and the results are shown in Fig. 6. The mixed mAb reagent demonstrated a high correlation with the Roche reagent (R² > 0.99) and an overall measurement bias of less than 5%. These findings highlight the excellent performance and clinical applicability of the mixed mAb reagent in diagnostic settings.

## Discussion

This study presents a proof-of-concept for a strategy in diagnostic reagent development by converting a high-performing polyclonal antibody into an optimized recombinant monoclonal antibody cocktail. We have developed the ApoA-I immunoturbidimetric assay based on mAb cocktail obtained through *de novo* sequencing from goat polyclonal antibodies. This approach delivers performance comparable to the original polyclonal reagent and shows good agreement with a leading commercial assay. As a result, it provides a defined and reproducible alternative to conventional reagent production methods.

A major challenge in transitioning from polyclonal to monoclonal-based turbidimetric assays has been balancing multi-epitope coverage with practical development constraints. Although polyclonal antibodies offer broad epitope recognition, their inherent variability hinders standardization [17, 18]. Conventional mAb strategies—using either a limited number of clones or complex murine cocktails—often fail to achieve sufficient avidity or encounter substantial development barriers [19, 20]. Our approach overcomes these limitations by directly mining the functional immunoglobulin repertoire of an immunized host. The *de novo* sequencing success rate of 22.3% for recovering functional variable regions, while reflecting the technical difficulty of reconstructing antibodies from a complex pool, supports the feasibility of the method and supplies a rich library for screening.

While previous reports sequenced human polyclonal antibodies using statistical pairing [7, 8], our goat-derived repertoire is substantially larger and more diverse [21]. To handle this complexity, we integrated co-elution profiling from non-reducing gels, MS abundance correlation, and experimental validation by recombinant expression and ELISA. This approach preserved the structural integrity and affinity of the original polyclonal response and enabled the identification of only four synergistic antibodies that, in combination, perform comparably to the parent polyclonal reagent.

The improved clinical performance of the four-mAb cocktail (clones 1#, 2#, 3#, and 6#) is likely attributable to synergistic binding to distinct, non-overlapping epitopes on ApoA-I. The essential contribution of mAb 3#, evidenced by a sharp performance drop upon its exclusion, indicates it recognizes a dominant and readily accessible epitope critical for immunoturbidimetric detection. In contrast, the antagonism observed with mAbs 4# and 5# suggests steric hindrance or competition for overlapping epitopes, reducing overall avidity. This rational epitope engineering yielded clear performance benefits: the cocktail showed strong correlation (R² > 0.99) with both the polyclonal reagent and the Roche commercial kit, a wider linear range, and a reduced hook effect. These properties likely arise from the optimized avidity and consistent, high-concentration formulation of the recombinant mAbs. By comparison, polyclonal reagents may include non-specific or low-affinity antibodies that constrain the dynamic range and introduce variability during purification and concentration. Moreover, the improved batch-to-batch consistency of our mAb cocktail underscores the fundamental advantage of recombinant reagents in sustaining long-term assay quality and reliability.

The development of a standardized ApoA-I assay addresses an important need in cardiovascular disease (CVD) management [22]. As the primary functional component of HDL, ApoA-I is a stronger inverse predictor of cardiovascular risk than HDL-C [23]. Accurate, reproducible ApoA-I measurement is therefore important for improved risk stratification. The reduced batch-to-batch variation of our mAb cocktail may enhance the reliability of long-term patient monitoring—for example, in tracking individual responses to lifestyle or pharmacologic interventions, where consistent measurement helps ensure that observed changes reflect genuine biological variation rather than analytical variability. The assay’s broad linear range and minimized hook effect also lower the frequency of sample re-runs due to out-of-range results, potentially improving laboratory efficiency and shortening turnaround times for clinical decision-making. By delivering a more robust and reliable tool, this assay could support more precise CVD risk evaluation and better-informed patient management.

Beyond ApoA-I, this work offers a scalable framework for modernizing polyclonal-based diagnostics. The strategy may be particularly suitable for targets requiring recognition of conformational epitopes, post-translational modifications, or complex antigenic surfaces. By combining de novo sequencing with functional screening, our approach enables the identification of complementary antibody panels from polyclonal repertoires. A promising next application is a multiplexed assay for the ApoA-I/ApoB ratio [24], a well-established CVD risk index, implemented with the same standardized methodology.

## Conclusions

This study establishes a practical strategy to overcome the batch variability of polyclonal antibodies in IVD. By applying *de novo* sequencing to a high-performing goat pAb, we developed a defined recombinant mAb cocktail against ApoA-I that retains broad epitope coverage without batch inconsistencies. In an immunoturbidimetric format, this four-mAb cocktail demonstrated excellent clinical correlation (*n* = 105; Passing-Bablok: y = 1.05x–0.07 g/L, r²=0.994), a wide analytical range (0.4–2.5 g/L), high sensitivity (LOD < 0.2 g/L), and superior precision (intra-/inter-batch CVs < 1% and < 2%). This work provides both a standardized assay for ApoA-I and a generalizable framework for developing consistent, next-generation IVD reagents.

## Electronic Supplementary Material

Below is the link to the electronic supplementary material.

Supplementary Material 1

## Data Availability

The datasets generated during and/or analyzed during the current study are available from the corresponding author on reasonable request.
