# Supplementary material for: From polyclonal to monoclonal: de novo sequencing of goat antibodies for a standardized ApoA-I immunoturbidimetric assay
Source: J Biol Eng. 2026 May 12;20:112. doi: 10.1186/s13036-026-00697-y (PMC13335323; doi:10.1186/s13036-026-00697-y)
Supplement: Supplementary file 1 — Supplementary Material 1 [file 13036_2026_697_MOESM1_ESM.docx]

**Supplementary Materials**

**
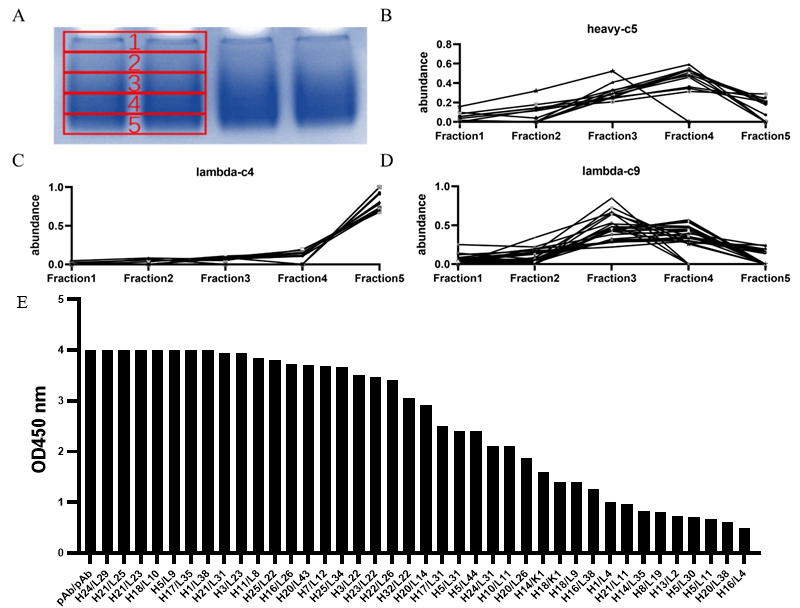
**

**Figure S1. Antigen-binding affinity of recombinant antibody pairs measured by indirect ELISA.** Seven recombinant mAbs, including the pair H5/L9, exhibited titers comparable to the original pAb, validating the effectiveness of the gel-based pairing strategy.


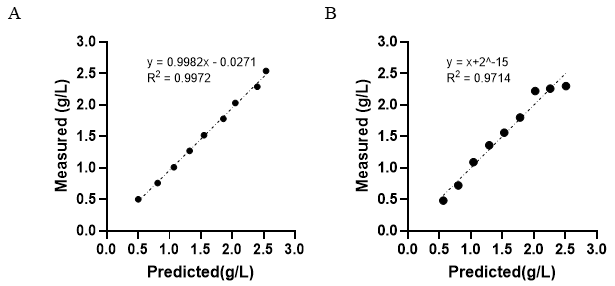


**Figure S2. Contrasting linearity performance of mAb and pAb reagents.** (**A**) The mAb reagent shows linear correlation across the 0.4–2.5 g/L range (R² = 0.9972). (**B**) The pAb reagent shows nonlinear response (R² = 0.9714).

**Table S1. Antibody Combinations and Settings**

| GROUP | 1# | 2# | 3# | 4# | 5# | 6# | PBS | R2 | Total |
| --- | --- | --- | --- | --- | --- | --- | --- | --- | --- |
| Mixed mAb Group A | 250μL | 250μL | 250μL | 250μL | 250μL | 250μL | 0μL | 500μL | 2000μL |
| Mixed mAb Group B | 0μL | 250μL | 250μL | 250μL | 250μL | 250μL | 250μL | 500μL | 2000μL |
| Mixed mAb Group C | 250μL | 0μL | 250μL | 250μL | 250μL | 250μL | 250μL | 500μL | 2000μL |
| Mixed mAb Group D | 250μL | 250μL | 0μL | 250μL | 250μL | 250μL | 250μL | 500μL | 2000μL |
| Mixed mAb Group E | 250μL | 250μL | 250μL | 0μL | 250μL | 250μL | 250μL | 500μL | 2000μL |
| Mixed mAb Group F | 250μL | 250μL | 250μL | 250μL | 0μL | 250μL | 250μL | 500μL | 2000μL |
| Mixed mAb Group G | 250μL | 250μL | 250μL | 250μL | 250μL | 0μL | 250μL | 500μL | 2000μL |

1#-6# represent six different monoclonal antibodies.

**Table S2. Interference from Triglycerides, Vitamin C, and Hemoglobin in the Assessment of the Mixed mAb Group and Poly-Ab Group Assays.**

| **Interference by triglycerides** | | | | | | | | | | | | | | | | | |
| --- | --- | --- | --- | --- | --- | --- | --- | --- | --- | --- | --- | --- | --- | --- | --- | --- | --- |
| Triglycerides（g/L） | | | 0 | | | 5 | | | 10 | | | 15 | | | 20 | | |
| Mixed mAb Group(mg/L) | | | 1.24 | | | 1.12 | | | 1.01 | | | 0.91 | | | 0.85 | | |
| Relative Deviation | | | / | | | -9.3% | | | -18.2% | | | -26.3% | | | -31.6% | | |
| Poly-Ab Group(mg/L) | | | 1.23 | | | 1.12 | | | 0.94 | | | 0.82 | | | 0.73 | | |
| Relative Deviation | | | / | | | -9.0% | | | -23.7% | | | -33.5% | | | -41.6% | | |
| **Interference by Vitamin** | | | | | | | | | | | | | | | | |  |
| **Vitamin C（g/L）** | | **0** | | | **0.25** | | | **0.50** | | | **0.75** | | | **1.00** | | |  |
| Mixed mAb Group(mg/L) | | 1.22 | | | 1.22 | | | 1.23 | | | 1.21 | | | 1.23 | | |  |
| Relative Deviation | | / | | | 0.4% | | | 0.8% | | | -0.4% | | | 0.8% | | |  |
| Poly-Ab Group(mg/L) | | 1.24 | | | 1.24 | | | 1.23 | | | 1.23 | | | 1.24 | | |  |
| Relative Deviation | | / | | | 0.4% | | | -0.4% | | | -0.4% | | | 0.0% | | |  |
| **Interference by Hemoglobin** | | | | | | | | | | | | | | | |  |  |
| **Hemoglobin（g/L）** | **0** | | | **2.5** | | | **5.0** | | | **7.5** | | | **10.0** | | |  |  |
| Mixed mAb Group(mg/L) | 1.25 | | | 1.22 | | | 1.22 | | | 1.20 | | | 1.23 | | |  |  |
| Relative Deviation | / | | | -2.4% | | | -2.4% | | | -4% | | | -1.6% | | |  |  |
| Poly-Ab Group(mg/L) | 1.23 | | | 1.27 | | | 1.27 | | | 1.27 | | | 1.28 | | |  |  |
| Relative Deviation | / | | | 3.3% | | | 3.3% | | | 3.3% | | | 4.1% | | |  |  |

**Table S3. Precision Testing of Mixed mAb Group and pAb Group for Three Batches**

| regent sample | Mixed mAb Group | | | | | | | | | Poly-Ab Group | | | | | | | | |
| --- | --- | --- | --- | --- | --- | --- | --- | --- | --- | --- | --- | --- | --- | --- | --- | --- | --- | --- |
|  | sample 1 | | | sample 2 | | | sample 3 | | | sample 1 | | | sample 2 | | | sample 3 | | |
|  | B1* | B2 | B3 | B1 | B2 | B3 | B1 | B2 | B3 | B1 | B2 | B3 | B1 | B2 | B3 | B1 | B2 | B3 |
| Reps1 | 1.99 | 1.99 | 1.99 | 1.01 | 1.01 | 1.02 | 0.60 | 0.62 | 0.60 | 1.95 | 2.05 | 1.99 | 1.02 | 0.99 | 1.01 | 0.65 | 0.57 | 0.60 |
| Reps2 | 1.94 | 1.99 | 1.97 | 1.02 | 1.01 | 0.99 | 0.59 | 0.62 | 0.60 | 2.00 | 2.05 | 1.94 | 1.05 | 0.99 | 1.02 | 0.65 | 0.57 | 0.59 |
| Reps3 | 2.03 | 1.99 | 1.93 | 1.04 | 1.01 | 0.99 | 0.59 | 0.62 | 0.60 | 2.04 | 2.05 | 2.03 | 1.05 | 0.99 | 1.04 | 0.66 | 0.56 | 0.59 |
| Reps4 | 2.01 | 1.98 | 1.99 | 1.03 | 1.00 | 1.00 | 0.59 | 0.62 | 0.60 | 1.98 | 2.04 | 2.01 | 1.05 | 0.99 | 1.03 | 0.66 | 0.57 | 0.59 |
| Reps5 | 1.97 | 1.97 | 1.98 | 1.04 | 1.00 | 1.00 | 0.59 | 0.62 | 0.61 | 2.02 | 2.04 | 1.97 | 1.06 | 0.98 | 1.04 | 0.64 | 0.56 | 0.59 |
| Reps6 | 1.95 | 1.98 | 2.00 | 1.03 | 1.00 | 1.01 | 0.59 | 0.62 | 0.60 | 2.01 | 2.05 | 1.95 | 1.06 | 0.99 | 1.03 | 0.64 | 0.57 | 0.59 |
| Reps7 | 1.98 | 1.97 | 1.97 | 1.03 | 1.01 | 1.00 | 0.59 | 0.62 | 0.60 | 2.02 | 2.04 | 1.98 | 1.05 | 0.98 | 1.03 | 0.64 | 0.57 | 0.59 |
| Reps8 | 2.00 | 1.98 | 1.98 | 1.04 | 1.01 | 1.00 | 0.60 | 0.61 | 0.59 | 2.09 | 2.05 | 2.00 | 1.07 | 0.98 | 1.04 | 0.65 | 0.57 | 0.60 |
| Reps9 | 1.98 | 1.98 | 1.98 | 1.05 | 1.01 | 1.01 | 0.59 | 0.61 | 0.60 | 2.01 | 2.04 | 1.98 | 1.04 | 0.99 | 1.05 | 0.65 | 0.56 | 0.59 |
| Reps10 | 1.95 | 1.97 | 1.98 | 1.04 | 1.01 | 1.00 | 0.60 | 0.62 | 0.60 | 2.00 | 2.04 | 1.95 | 1.05 | 0.99 | 1.04 | 0.65 | 0.57 | 0.60 |
| Mean | 1.98 | 1.98 | 1.98 | 1.04 | 1.01 | 1.00 | 0.59 | 0.62 | 0.60 | 2.01 | 2.05 | 1.98 | 1.05 | 0.99 | 1.04 | 0.65 | 0.57 | 0.59 |
| SD | 0.03 | 0.01 | 0.02 | 0.01 | 0.00 | 0.01 | 0.00 | 0.00 | 0.00 | 0.04 | 0.01 | 0.03 | 0.01 | 0.00 | 0.01 | 0.01 | 0.00 | 0.00 |
| intra-batch CV% | 1.45% | 0.41% | 0.96% | 1.11% | 0.48% | 0.92% | 0.81% | 0.68% | 0.79% | 1.83% | 0.26% | 1.45% | 1.27% | 0.49% | 1.11% | 1.14% | 0.85% | 0.81% |
| Mean | 0.94% | | | 0.84% | | | 0.76% | | | 1.18% | | | 0.96% | | | 0.93% | | |
| inter-batch CV% | 1.00% | | | 1.61% | | | 1.92% | | | 1.87% | | | 2.83% | | | 5.85% | | |

Sample 1 represents high-value samples; Sample 2 represents medium-value samples; Sample 3 represents low-value samples; B1-B3 represents Batch 1 to Batch 3.

**Table S4. Linearity validation of the mAb and pAb reagents.**

| The mAb reagents | Measure  (g/L) | Predicted  (g/L) | Relatived  (g/L) | Absolute Deviation (g/L) | The pAb reagents | Measure  (g/L) | Predicted  (g/L) | Relatived  (g/L) | Absolute Deviation  (g/L) |
| --- | --- | --- | --- | --- | --- | --- | --- | --- | --- |
| H*^a^* | 2.54 | 2.54 | 0.00% | 0 | H | 2.30 | 2.51 | -8.23% | 0.21 |
| 7/8H + 1/8L | 2.29 | 2.4 | 5.03% | -0.11 | 7/8H + 1/8L | 2.26 | 2.26 | -0.12% | 0.00 |
| 3/4H + 1/4L | 2.03 | 2.05 | 0.99% | -0.02 | 3/4H + 1/4L | 2.22 | 2.02 | 9.96% | -0.20 |
| 5/8H + 3/8L | 1.78 | 1.86 | 4.51% | -0.08 | 5/8H + 3/8L | 1.80 | 1.78 | 1.39% | -0.02 |
| 1/2H + 1/2L | 1.52 | 1.55 | 1.97% | -0.03 | 1/2H + 1/2L | 1.56 | 1.53 | 1.85% | -0.03 |
| 3/8H + 5/8L | 1.27 | 1.32 | 4.35% | -0.05 | 3/8H + 5/8L | 1.36 | 1.29 | 5.59% | -0.07 |
| 1/4H + 3/4L | 1.01 | 1.07 | 5.94% | -0.06 | 1/4H + 3/4L | 1.09 | 1.04 | 4.37% | -0.05 |
| 1/8H + 7/8L | 0.76 | 0.81 | 6.62% | -0.05 | 1/8H + 7/8L | 0.72 | 0.80 | -10.07% | 0.08 |
| L | 0.5 | 0.5 | 0.00% | 0 | L | 0.48 | 0.56 | -14.72% | 0.08 |

The linearity of the assay was validated over the range of 0.40 to 2.50 g/L, requiring a correlation coefficient (r) of ≥ 0.990.

The linearity deviation was defined as an absolute deviation of ≤ 0.10 g/L within [0.40, 1.00) g/L and a relative deviation of ≤ 10% within [1.00, 2.50] g/L.

*^a^* H: high-value sample; L: low-value sample
